# Quantifying periodicity in omics data

**DOI:** 10.3389/fcell.2014.00040

**Published:** 2014-08-19

**Authors:** Cornelia Amariei, Masaru Tomita, Douglas B. Murray

**Affiliations:** Institute for Advanced Biosciences, Keio UniversityTsuruoka, Japan

**Keywords:** periodicity tests, waveform analysis, metabolic oscillation, metabolomics, flow cytometry

## Abstract

Oscillations play a significant role in biological systems, with many examples in the fast, ultradian, circadian, circalunar, and yearly time domains. However, determining periodicity in such data can be problematic. There are a number of computational methods to identify the periodic components in large datasets, such as signal-to-noise based Fourier decomposition, Fisher's *g*-test and autocorrelation. However, the available methods assume a sinusoidal model and do not attempt to quantify the waveform shape and the presence of multiple periodicities, which provide vital clues in determining the underlying dynamics. Here, we developed a Fourier based measure that generates a de-noised waveform from multiple significant frequencies. This waveform is then correlated with the raw data from the respiratory oscillation found in yeast, to provide oscillation statistics including waveform metrics and multi-periods. The method is compared and contrasted to commonly used statistics. Moreover, we show the utility of the program in the analysis of noisy datasets and other high-throughput analyses, such as metabolomics and flow cytometry, respectively.

## Introduction

Cellular network dynamics are excitable and inherently non-linear, properties that stem from the multitude of feedback and feedforward loops involved in biological processes (Lloyd, 2008). These systems form an intimate feedback with the environment to generate the dynamic phenotype of the cell (e.g., oscillation/pulsing, bursting bistability) (Sobie, 2011; Levine et al., 2013). The feedback and feedforward systems have drastically different time scales that vary over several orders of magnitude (Aon et al., 2004), from the annual migration patterns found in monarch butterflies (Kyriacou, 2009), to the second oscillation of cardiomyocytes in one's heart (Aon et al., 2004). While our understanding of each time scale increases daily, the interaction between different dynamical processes remains poorly characterized. Understanding the dynamical interactions between time scales are key to understanding the complex phenotypes of embryogenesis (Kageyama et al., 2012), circadian biology in disease (Gibbison et al., 2013), and psychology (Salvatore et al., 2012; Salomon and Cowan, 2013).

Our studies using frequently sampled data from yeast and cardiomyocytes showed that the time-structure is highly organized (Aon et al., 2008) and had the properties of a fractal over five orders of magnitude, indicative of harmonic entrainment in cellular processes. Moreover, cellular energetics and especially mitochondrial activity play defining roles in rapidly shaping cellular dynamics. Thousands of data points are required to study these orders of magnitude (Sasidharan et al., 2012c). However, analysing multiperiodicity in less frequently sampled data (under 100 data points) remains difficult (de Lichtenberg et al., 2005), and these are the kind of datasets commonly used for time-series expression or metabolic studies. Perhaps one of the best characterized synchronous oscillatory systems in this regard is the precisely controlled continuously cultured yeast. When environmental cues are removed, the resulting output in respiratory state (readily measured by residual dissolved oxygen measurements) is often a stable oscillatory or homeodynamic state (Lloyd et al., 2001; Lloyd and Murray, 2005, 2006, 2007; Johnson and Egli, 2014). This has been shown to be multi-oscillatory (Aon et al., 2008; Sasidharan et al., 2012c), to have period doubling events (Salgado et al., 2002; Klevecz and Li, 2007) caused by perturbation, and has multiple omic and high-throughput datasets available (Klevecz et al., 2004; Li and Klevecz, 2006; Murray et al., 2007; Sasidharan et al., 2012b,c). These properties make it an ideal model system for multi-scale dynamical studies.

Generally, analysis methods are restricted to the period of interest, such as the perturbation length or oscillation period, and the sampling frequency limits the use of many powerful time-series analysis tools (Dowse, 2007). Techniques such as autocorrelation (Yamada and Ueda, 2007) and Fourier transform (Yamada and Ueda, 2007; Lehmann et al., 2013) rely on targeting a particular frequency, and can be prone to generating false calls due to frequency changes and multi-oscillators. Singular Value Decomposition (SVD)/Principal Component Analysis (PCA) generally assumes that the largest variances are the most interesting (neglecting subtle effects), and also does not allow for the use of *a priori* dynamical knowledge to the analyses (Wang et al., 2012). Furthermore, it is difficult to assign meaning to the contributions of each time-series to the components (Raychaudhuri et al., 2000; Alter et al., 2003). Wavelets analyses are powerful, however the data density required makes it difficult to apply to the low-density time-series data generated from high-throughput experiments (Klevecz and Murray, 2001; Song et al., 2007; Prasad and Bruce, 2008; Sasidharan et al., 2012c).

Here, we introduce a tool that expands on the signal-noise (SN) ratio approach (Yamada and Ueda, 2007; Machné and Murray, 2012), by calculating the SN ratio of each frequency and then uses this to generate a model waveform whose goodness of fit to the original data is determined using coefficient of determination (*R*^2^). A user-specified significance or SN ratio cut-off determines the powers to use in constructing the model. We illustrate its utility using previously published data.

## Materials and methods

### Frequency model

Given a time-series of *N* points *x*_1_, *x*_2_,…, *x_N_*, the corresponding discrete Fourier transform (DFT), as a series of complex numbers *X*_0_, *X*_1_, …, *X*_*N*−1_, is given by the formula:

$$X_{k}=\sum_{n\;=\;0}^{N-1}x_{n}{\text{e}}^{-i2\pi k\frac{n}{N}},k=0,\ldots ,N-1,$$

where *X_k_* represents the component of *k* cycles per time-series.

The component of frequency 0 (*X*_0_) is used to calculate the mean value of the time-series, referred to as the DC component:

$$DC=\frac{\left|X_{0}\right|}{N}$$

By the nature of the DFT, the remaining components *X*_1_, *X*_1_, …, *X*_*N*−1_ are mirrored:

$$X_{k}=X_{N-k},k=1,\ldots ,M,M=\left\lfloor \left(N-1\right)/2\right\rfloor ,$$

therefore, all further calculations are performed on the first half of these components.

The peak-to-peak amplitude *A_k_* for each frequency *X_k_* is given by the formula:

$$A_{k}=\frac{4}{N}*\left|X_{k}\right|,\;k=1,\ldots ,M$$

The SN ratio (Yamada and Ueda, 2007) represents the ratio between the amplitude of the target signal and the average amplitude of noise (i.e., the average amplitude of all other frequencies):

$$SN_{k}=\frac{\left(M-1\right)*A_{k}}{\left(\sum_{n\;=\;1}^{M}A_{n}\right)-A_{k}},\;k=1,\ldots ,M$$

For the construction of the model, if no target frequency is specified (untargeted mode), the algorithm removes all frequencies that are considered noisy (i.e., that do not pass the arbitrary *sn* threshold). Thus, a filtered set of signals *Xf_k_* is calculated by removing the frequencies with a SN ratio below the *sn* threshold, while preserving the DC component:

$$Xf_{k}=\{\begin{array}{ll} X_{k} & \text{if }SN_{k}>sn\text{ or }k=0\\ 0 & \text{otherwise} \end{array},\;k=0,\ldots ,N-1$$

If a target frequency *ta* is specified (targeted mode), the intent of the algorithm is to preserve the harmonics of the specified frequency that oscillate, including possible temporal drift into the frequency *ta* − 1 and its harmonics, but to remove all frequencies that have an oscillation stronger than the target frequency, or are too noisy (below the *sn* threshold). Thus, only the frequencies *ta* − 1 and higher are kept, only if they have a lower SN ratio than*X_ta_* and only if they pass the *sn* threshold (also preserving component 0, i.e., the mean):

$$Xf_{k}\;=\;Xf_{N-k}\;=\;\{\begin{array}{ll} X_{k} & \text{if}(SN_{k}>sn\text{ and }SN_{k}\leq SN_{ta}\;\\ & \text{and}k\geq \left(ta-1\right))\text{or }k\text{​}=\text{​}0\\ 0 & \text{otherwise} \end{array},\text{​}k=0,\ldots ,M$$

If *N* is even, the middle component *Xf*_*M* + 1_ is also set to 0.

As it can be seen, if the SN of the targeted frequency does not pass the *sn* threshold, all components are removed (resulting in a flat line). If the user-specified cut-off is given as a *P*-value, the *sn* cut-off is the corresponding SN ratio at the given *P*-value.

In all cases, the user can override these filters by manually specifying components to be omitted. The filtered waveform is reconstructed by the inverse DFT:

$$xf_{k}=\frac{1}{N}\sum_{n\;=\;0}^{N-1}Xf_{n}{\text{e}}^{i2\pi k\frac{n}{N}},k=0,\ldots ,N-1$$

**Figure 1:**
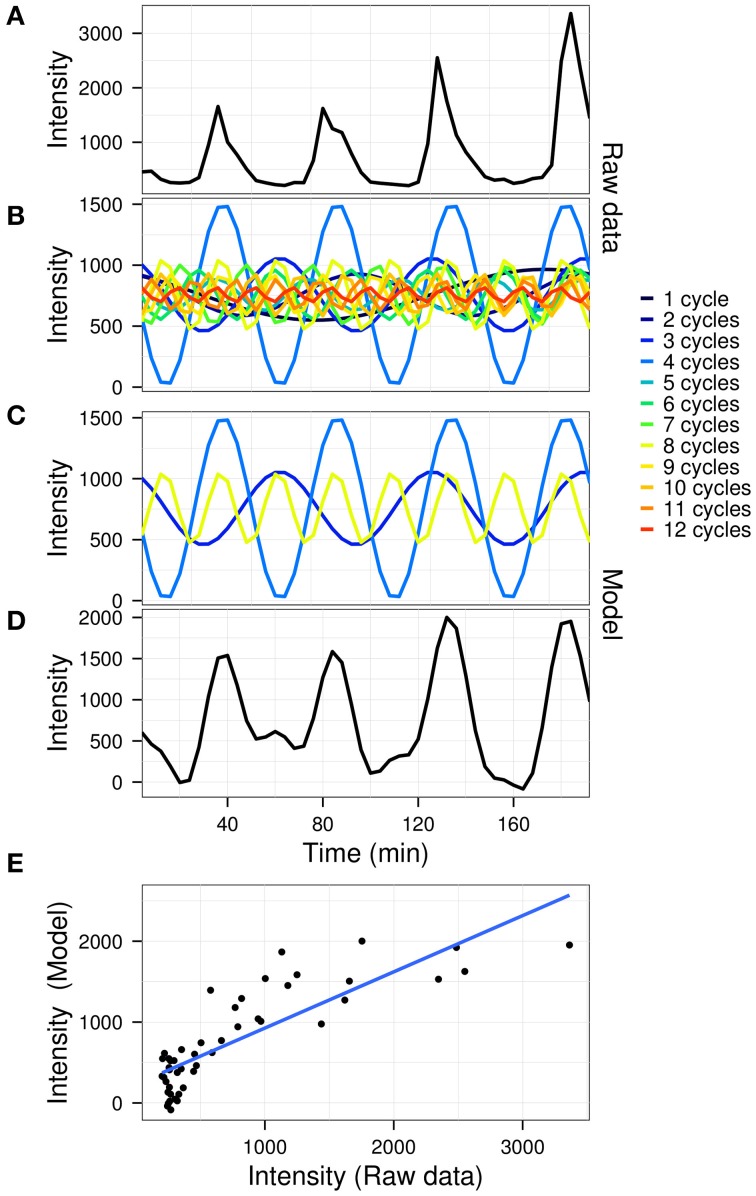
**A graphical representation of the model construction in untargeted mode.** The raw gene expression time-series of gene YAL067C (**A**; arbitrary fluorescence units) is first decomposed by fast Fourier transform **(B)**. The significant powers which comprise the signal **(C)** are then recomposed to produce the model **(D)**. A linear fit is then used to determine the coefficient of variation (**E**; *R*^2^ = 0.695) for the data **(A)** vs. the model **(D)**.

The goodness of fit between the model and the original data was calculated using *R*^2^ values. A graphical outline of the algorithm is presented in Figure 1, using the gene expression time-series (dataset described below) for yeast gene YAL067C (the first oscillator in the dataset).

The algorithm was developed in R (R Core Development Team, 2008) and is called waveform. The main parameters passed are the cut-off method (SN ratio or its *P*-value) and cut-off threshold (default to 2 and 0.05, respectively). The statistics necessary for full characterization of the Fourier components (DC, amplitude, and angle) are calculated by the underlying function oscilGet, which also generates statistics on autocorrelation (Venables and Ripley, 2002), Ljung-Box test (Ljung and Box, 1978), Oscillation Strength (Murray et al., 2007), and Fisher's exact *g*-test (Ahdesmäki et al., 2005).

The significance calculation method can also be specified, i.e., “model” for log normal distributions or a number of iterations for a permutation-based statistic (10,000 is the default). The model-based significance calculation first generates a normal probability distribution from 10,000 random samples, using the standard deviation of the analyzed dataset or a user-specified standard deviation. Next, the statistics for signal-noise ratio, oscillation strength, and/or autocorrelation on the model data are generated. The standard deviation and mean of the target statistics are used to generate a model distribution for each statistic, and the significance is then calculated from the experimental data and the model statistics' upper tail. For this approach to work, the distribution of the dataset should be checked carefully for the normality of the majority of the data. The distribution is sensitive to experimental noise (i.e., limits of experimental determination can result in skewed tails which alter the standard deviation of the dataset), and this can be accounted for prior to analysis by passing the standard deviation of the log-normal subset of the dataset onto the algorithm (see supplemental package, data manuals for examples).

If the distribution deviates significantly from log normality, then the permutation approach can be used (with at least 10,000 iterations, to avoid high false discovery rates). The rows of the data matrix are permuted by the specified number of iterations, and *P*-values are defined as the ratio between the number of times the statistic of the permutation was greater than the statistic of the original data and the number of iterations. This is computationally intensive and one can specify the number of slaves (nSlaves) for multicore systems. Lower iteration numbers increase the false discovery rates; to address this, the optimal iteration number can be determined with existing R packages, such as fdrtool (Strimmer, 2008). For a *P*-value of 0.01 we found 10,000 iterations to give an acceptable false discovery rate (0.0043).

The supplemental R-package waveform contains full details, examples and the data used, and uses three main commands; waveform, oscilGet, and DFT. DFT is a wrapper for the default fast Fourier transform of R (fft), which uses a Mixed-Radix algorithm (Singleton, 1969). The package can be installed using the following command:

R CMD INSTALL waveform_1.0.1.tar.gz

The package requires the standard R packages: GeneCycle, matrixStats, foreach, doSNOW, fdrtool, iterators, snow, and e1071. Updates will be available for download from http://oscillat.iab.keio.ac.jp.

### Experimental data

We used three published experimental datasets for this study. To illustrate the general uses of the algorithm, we used a highly oscillatory transcriptome (Affymetrix GeneChip®) experiment from metabolically synchronous continuous yeast cultures which were perturbed with the monoamine oxidase inhibitor, phenelzine (Li and Klevecz, 2006). This consisted of 4 oscillation cycles (48 samples, taken every 4 min) and was perturbed after 48 min (sample 12). As an example of a noisy dataset with unknown biological and technical peaks, we used a metabolome time-series, containing unidentified peaks, from similar metabolically synchronous cultures, comprising of 2 oscillation cycles (20 samples, taken every 4 min) that was not perturbed (Sasidharan et al., 2012b). Finally, we used a dataset with absolute quantified values, a set of propidium Iodide DNA stained flow cytometry yeast samples (Klevecz et al., 2004), which consisted of four unperturbed cycles (60 samples, taken every 2.5 min) and was aligned to the peaks observed at G_1_ and G_2_. It is important to note that all the data shown here are raw and have not been normalized.

The distributions of these datasets (once zero and noisy low abundance measurements had been filtered) all approximated to a log normal distribution, thus we used the model-based approach for all analyses.

## Results

### The SN ratio outperforms other tested oscillation metrics

**Figure 2:**
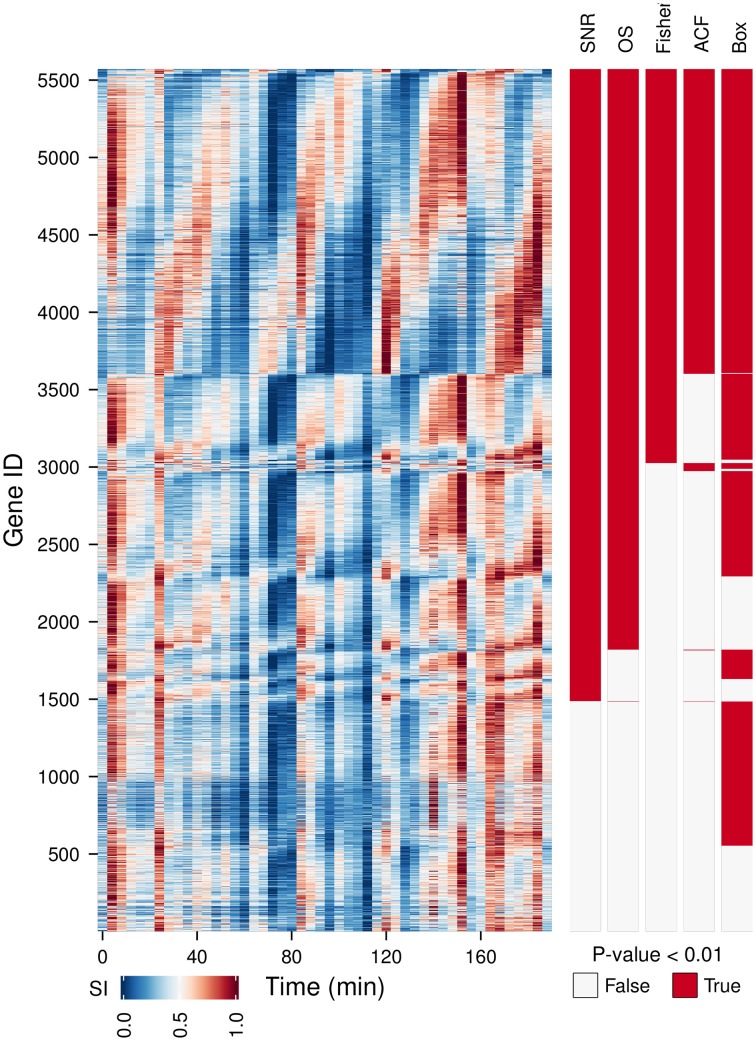
**A comparison between different oscillation tests.** An oscillatory transcriptome dataset (Li and Klevecz, 2006) containing 5570 gene expression profiles during a perturbation experiment (injection of 1 mM phenelzine at min 48) was used to test five oscillation metrics: signal-to-noise ratio (SN ratio) (Yamada and Ueda, 2007; Lehmann et al., 2013), oscillation strength (OS) (Murray et al., 2007), Fisher's exact *g*-test (Fisher) (Ahdesmäki et al., 2005), autocorrelation function (ACF) (Venables and Ripley, 2002), and the Ljung box-test (Box) (Ljung and Box, 1978). Gene IDs were first sorted according to common hits (*P*-value < 0.01 for SN ratio, OS, Fisher on period 4 and for ACF and Box on lag 12) and then by the phase angle of the dominant frequency of the data (4 cycles). The temporal profile of each gene was scaled (SI) for visualization purposes.

**Figure 3:**
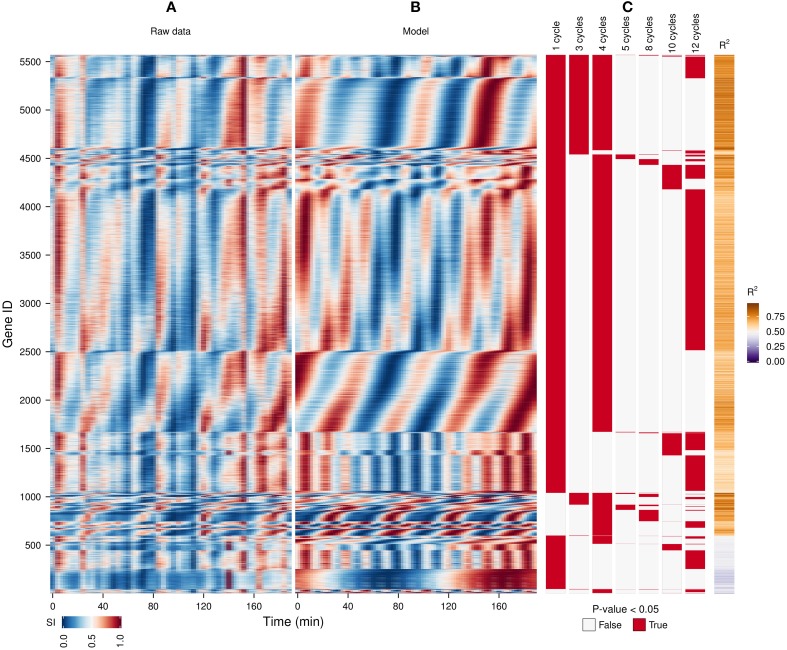
**Identification of expression cohorts using major spectral powers.** The waveform algorithm was used on a raw transcriptome dataset **(A)** taken during a perturbation experiment (injection of 1 mM phenelzine at min 48) (Li and Klevecz, 2006) to generate a model using the default settings (**B**; *R*^2^ values shown in sidebar). Genes were first sorted according to the presence of all oscillatory components identified in the dataset after the *P*-value cut-off of 0.05 **(C)**, and then by the phase angle of the dominant frequency of the data (4 cycles). The genes profiles with a *R*^2^ < 0.5 are shown at the bottom. The temporal profile of each gene was scaled (SI) for visualization purposes.

We have tested the capabilities of 5 oscillation tests on a time-series microarray gene expression dataset (Li and Klevecz, 2006) containing 5570 gene expression profiles. A comparison between the oscillators with the main period of the dataset (4 cycles) detected (Figure 2, OS, SN ratio, Fisher's exact *g*-test, ACF, Box) shows a good agreement between methods for 35.8% of the genes, providing a gold standard for visualizing discrepancies between tests. As Fisher's exact *g*-test (the most conservative approach), SN and OS are based on similar methods, these provided the best agreement on the 4 cycle frequency. Fisher's exact *g*-test however only reports the dominant frequency in the dataset and was not useful for further characterization of multi-periodicity and period lengthening. OS and SN ratio detected major powers in profiles with strong multiperiodicities better. ACF failed to pick up clear oscillatory signals. Whereas, Ljung-Box analysis called many non-oscillatory time-series, probably due to the low amplitude, but significant 12 cycle frequency (Figure 3). Therefore, our algorithm was based on the SN ratio.

### Determination of frequencies and phase relationships during a perturbation

**Figure 4:**
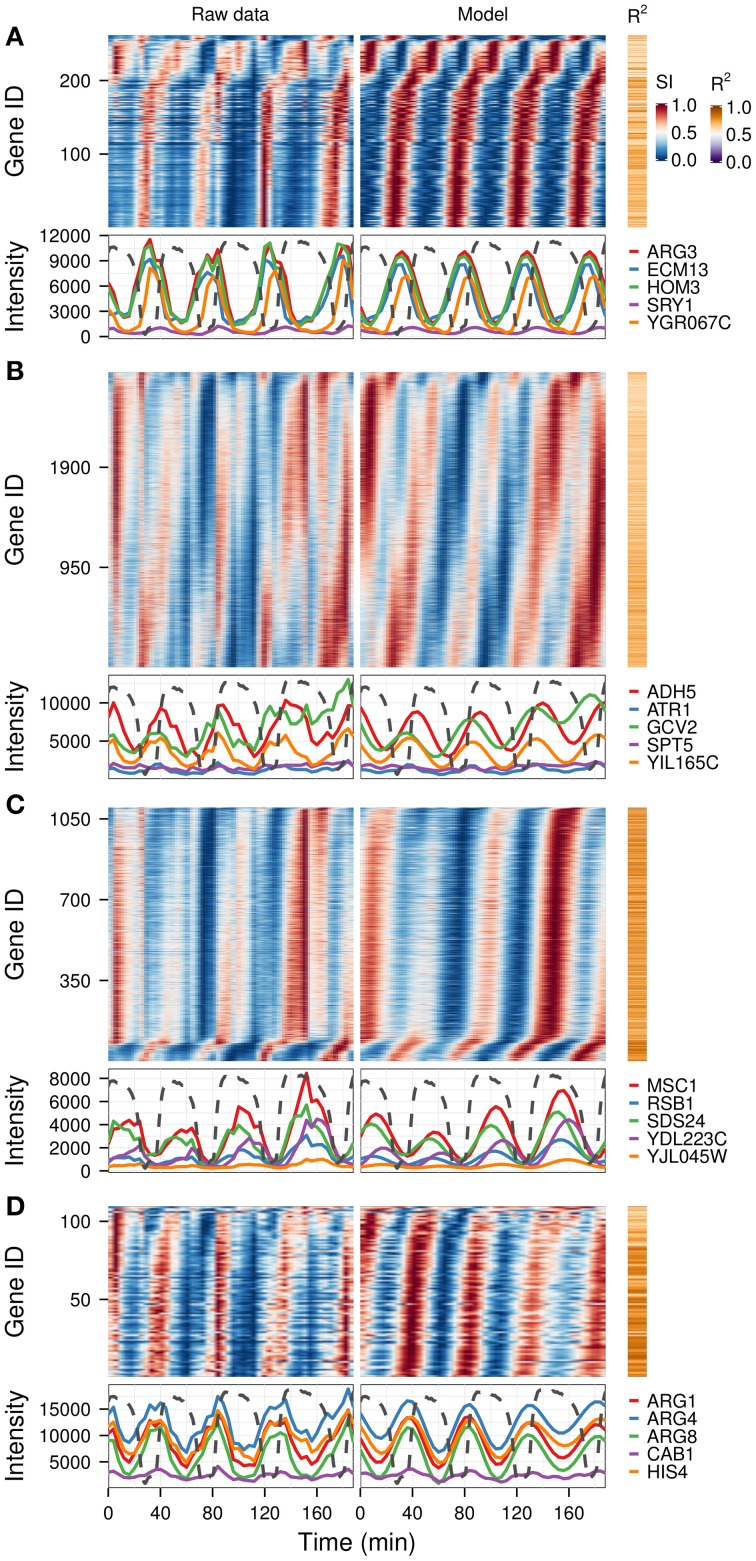
**Identification of differential responses to perturbation.** Based on the analysis presented in Figure 3, four gene cohorts with differential responses were identified based on the presence of the spectral components of interest (1, 3, 4, 5). Genes showing a 4-cycle oscillation and no period drift (no 3 and 5 components) were separated into genes with no major trend over the experiment **(A)**, and those that had a response to the experiment **(B)**. Genes with 3-cycle **(C)** and 5-cycle components **(D)** are shown separately. The top 5 genes with the highest *R*^2^ in each cohort are shown in the bottom panel of each graph, against the corresponding dissolved oxygen (DO) trace (dotted lines), which was scaled to the range of the plot. The perturbation agent (phenelzine, 1 mM) was injected at min 48. The temporal profile of each gene was scaled (SI) for visualization purposes.

A previous study of this gene expression dataset, which used the Fourier spectra for clustering (Machné and Murray, 2012) has successfully identified biologically-coherent clusters, but concentrated on characterizing the phase-relationship of gene expression with respect to the respiratory oscillation. However, the analysis of the dataset with the waveform algorithm, untargeted, with default parameters, indicated that several major frequencies occurred (1, 3, 4, 5, 8, 10 and 12 cycles, 91.3%, 21%, 79.4%, 2.2%, 4.5%, 10.2%, 54.3% genes, respectively). Visualization of cohorts obtained by grouping genes based on the presence of these periodicities in their filtered spectra and the *R*^2^ values pointed to components 1, 3–5 as sufficient to discriminate between the major expression patterns (Figure 3). To exemplify different responses to the perturbation, we selected 4 cohorts. The first one comprises of genes who had no significant response to the drug (only significant frequency was 4 cycles; Figure 4A, 4.7% of genes), and was highly enriched in genes involved in cytosolic ribosomal assembly and sulfur amino acid processes (Table S1). Cohort 2 represents genes that had a significant response to the chemical perturbation, but did not show a strong increase or decrease in amplitude (significant 1 and 4 cycle, but not significant 3 and 5 cycle components; Figure 4B, 50% of genes). This cohort was enriched in translation (Table S1). Cohort 3 contained genes with significant 3 and 4 cycle components (Figure 4C; 20% of genes). The mRNA abundances of these genes were influenced by the period lengthening effects of the drug and show the intensities drop immediately after perturbation. However, they increase in intensity as the experiment progresses so that the final intensities on the perturbed long period cycles are higher than the initial cycle. Cohort 3 was highly enriched in mitochondrial and catabolic processes (Table S1). Cohort 4 comprised a combination of significant 4 and 5 cycles (Figure 4D; 2% of genes). The mRNA abundance of these genes showed a decrease in oscillation amplitude during the experiment's progression and the 5 cycle periodicity is due to the first 2 cycles which have higher amplitudes for these genes. Ontology enrichment showed that cohort 4 was primarily involved in anabolic processes, with the top 5 genes involved in the Arginine, Coenzyme A, and Histidine biosynthetic pathways. As 80% of the genes peak during the phase of high residual dissolved oxygen (Figure 4; gray dotted lines), the phase relationships between the cohorts was not evenly distributed. The maximum of cohort 1 was skewed toward the phase of low DO, cohort 2, representing the majority of the dataset (Li and Klevecz, 2006), peaked right after the transition between low and high DO, cohort 3 was almost exclusively expressed during the high DO phase and cohort 4 was skewed toward the end of the low DO phase. Further refinement of this classification based on the phase-angle of the main periodicity leads to similar results as the previous clustering-based approaches (Machné and Murray, 2012), exemplifying a way to significantly reduce the size of a dataset, in our case from 48 variables (time-points) to 5 (4 spectral components and the phase angle of the major component).

### Waveform analysis can extract information from complex and noisy datasets

**Figure 5:**
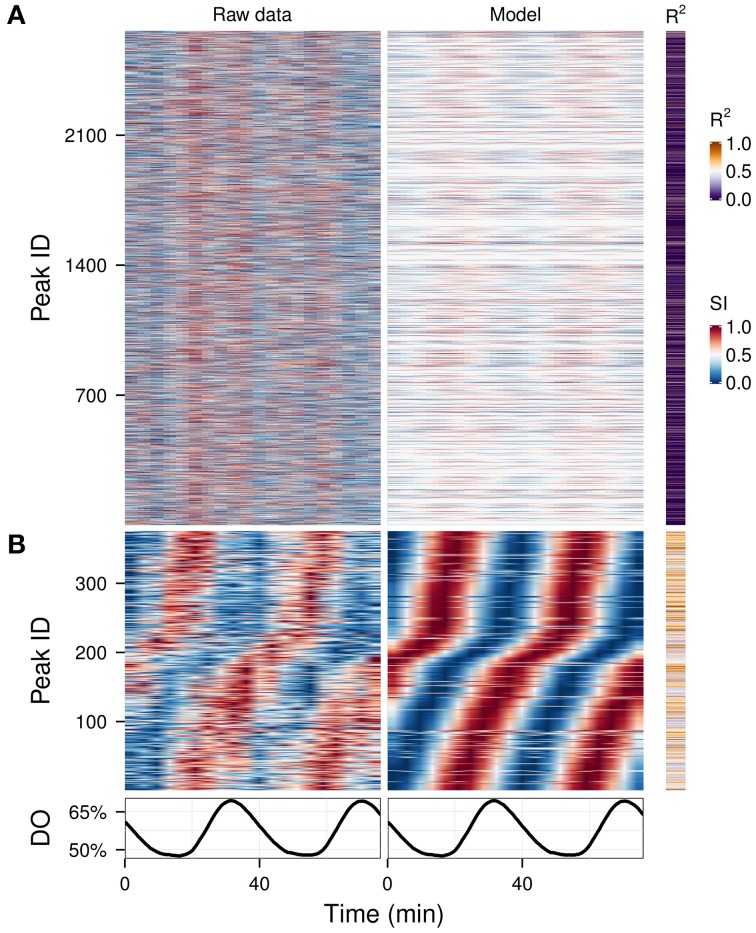
**Exploratory examination of a noisy time-series metabolomics dataset.** A time-series dataset of unidentified CE-MS peaks (**A**, left panel) (Sasidharan et al., 2012b) was filtered using the waveform algorithm with default cut-off and targeting the dominant frequency of the data (2 cycles; **A**, right panel). The statistically significant peaks based on the coefficient of determination (*R*^2^) are shown in **(B)**. Peak IDs were sorted using hclust (stats package in R) (Murtagh, 1985) with the euclidean distance and Ward's method (Ward, 1963) in **(A)** and by the phase angle of the 2-cycle component in **(B)**. The corresponding dissolved oxygen (DO) trace during the experiment is show in bottom panel. The temporal profile of each peak was scaled (SI) for visualization purposes.

Hybridizations on microarrays produce data in which most of the signal should be biological in origin. However, mass spectrometry is much noisier, because many peaks are caused by environmental contamination, caused by column components or degradation. We analyzed a complex data matrix from a metabolomics study containing 2661 peaks (Sasidharan et al., 2012b) on which usual clustering could not easily discriminate between technical and biological signals (Figure 5A, left panel). We ran the waveform algorithm targeting the oscillation period (2 cycles, *P*-value cut-off 0.05), thus keeping only the peaks which had a significant 2-cycle component and removing all masking frequencies. The resulting waveforms, in which time-series with no significant 2-cycle components were reduced to flat signals, making the oscillators apparent throughout the dataset (Figure 5A, right panel), and after removing peaks with poor fit (*P*-value > 0.01), 375 potential biological signals were identified (Figure 5B), demonstrating a quick and effective method for exploratory metabolomics.

### Data processing while preserving phase angles and amplitudes

**Figure 6:**
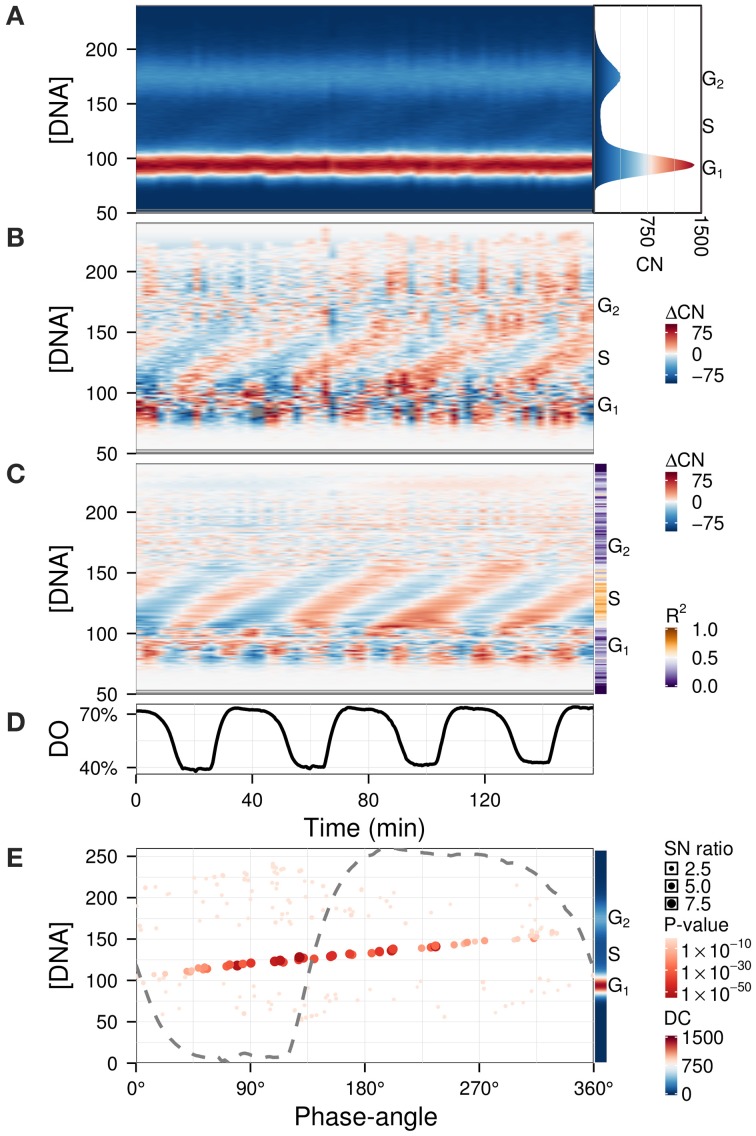
**Identification of phase-relationships in a flow cytometry dataset (Sasidharan et al., 2012a).** Each datapoint represents the number of cells (CN) in a particular DNA intensity bin (peak propidium iodide channel) (Klevecz et al., 2004). These were aligned and scaled according to the G_1_ and G_2_ peaks (**A**; histogram of the average CN over the time-series is shown in right panel). Residuals **(B)** were calculated by subtracting the average CN over the time-series, and were filtered using the waveform algorithm (**C**; *R*^2^ values shown in sidebar). The corresponding dissolved oxygen (DO) trace during the experiment is shown in **(D)**. The major component (4 cycles) was characterized by the phase-angles with respect to the respiratory oscillation and SN ratio at each DNA concentration **(E)**; the DC component is shown in the sidebar. The dashed gray line represents the DO trace over one cycle, scaled to the range of the panel. Phase-angles 0°/360° represent the minimum of the DO rate in each respiratory cycle.

The previous examples contained qualitative measurements, therefore amplitudes were relative values. To illustrate the use of Fourier decomposition in denoising data while preserving the temporal structure, we used a quantitative flow cytometry time-series dataset (Figure 6A) (Sasidharan et al., 2012a). The purpose of the analysis was to identify the phase-relationship, significance of oscillation and duration of the DNA division cycle. While subtracting the background (Figure 6B) already reveals the main patterns, information such as the precise timing of DNA replication with respect to the respiratory oscillation and the amplitude in the S-phase regions are not trivial to extract. The waveform model was used to accentuate the regions of interest by using an untargeted approach with the default parameters (Figure 6C). Interestingly, S-phase was shown to be a linear time series that continues throughout the respiratory cycle, starting during the phase where residual dissolved oxygen was lowest (Figure 6E), which was earlier than previously reported (Klevecz et al., 2004). This could only be observed when we filtered out the contaminating frequency components from the much larger G_1_ and G_2_ cell cycle phase peaks. This analysis may resolve observed differential timings of mid S-phase found for different oscillation periods (Slavov et al., 2011; Amariei et al., 2013).

## Discussion

We present a set of tools that can be used to dissect oscillatory data, with or without a perturbation. It can be used for any data matrix that is from an oscillatory system, such as transcriptomic, metabolomics, and proteomic, as well as other single or high-throughput measurements. We show its utility in highlighting biological processes such as S-phase (Figure 6), a separation of biologically relevant signals from noisy metabolomic data (Figure 5) and delineating perturbation effects in a drug treatment experiment (Figures 3, 4). Additionally, analyses on this perturbation separated events spanning different time-scales, i.e., the long perturbation event (10 h) (Li and Klevecz, 2006), the oscillation (40 min) and sub-events that may be related to changes in cofactor abundance (10–15 min) (Sasidharan et al., 2012c). For the yeast oscillatory system, it is relatively easy to cross-correlate time series taken in different laboratories, form different oscillation periods, using data taken months (or even years apart) by adjusting the phase angle with respect to a reference point on the residual dissolved oxygen data (Murray et al., 2003; Lloyd and Murray, 2007; Machné and Murray, 2012), thus opening up a wealth of data to the experimenter.

A common issue that arises when dealing with large datasets is the excessive requirements for computational power and memory for calculating distance matrices, which limits clustering methods. Filtering spectral components (Figures 3, 4) can be an effective way of reducing the complexity of the dataset before clustering. Indeed, the majority of the ontology enrichments previously observed by Machné and Murray (2012) were also reconstituted in the frequency analysis reported here.

Normalization of oscillatory time-series datasets is often a difficult task due to lack of an internal, biological set of non-oscillating references, and the steps taken can alter the data structure significantly (Lehmann et al., 2013). If subjected to standard array-to-array normalization methods which include an alignment to the mean of individual arrays, the phase-angles of expression in Figure 3 would be significantly skewed due to higher mRNA abundance in one phase of the respiratory oscillation. Even the seemingly noisy minor peaks that occur every 3-4 samples (the 12-cycle component which is found in over half of the transcripts) may be biological, as they coincide with the triphasic patterns of NAD(P)H fluorescence occurring during the yeast respiratory oscillation (Sasidharan et al., 2012c). Furthermore, attempting to normalize the metabolite dataset in Figure 5 using internal standards deteriorated the 2-cycle oscillatory signal, indicating that biological signals were less noisy than the external controls. Therefore, aggressive normalization of such periodic data should generally be avoided. However, when normalization is necessary, the presented algorithm can be used to identify a subset of least-oscillatory biological features on which normalization can be carried out, and the fitting parameters thus obtained can then be used to normalize the rest of the dataset, while preserving its temporal profile (Calza et al., 2008; Machné and Murray, 2012).

The methods presented here can readily be used to analyse short time-series data taken in triplicates, by concatenating the triplicate series to obtain a pseudo-waveform spanning 3 “pseudo-cycles.” However, one prerequisite and major limitation for general Fourier based approaches is that the analyzed dataset must be sampled at equal time intervals. If the time-series in question has uneven sample times (e.g., 0, 5, 15, 30, 60, 120, 480 min) it may still be possible to utilize the algorithm on the pseudo-waveform constructed from the triplicates, by applying the appropriate data window to adjust the monotonically increasing or decreasing profiles (such as Hamming or Hanning (Oppenheim et al., 1999); already implemented in the waveform package), as these are prone to spectral leakage (Lyon, 2009). The resulting data would then be readjusted to the original timing. Thus, future developments of the algorithm will be its application to certain non-oscillatory and non-equally sampled datasets.

### Conflict of interest statement

The authors declare that the research was conducted in the absence of any commercial or financial relationships that could be construed as a potential conflict of interest.

## Supplementary material

**Supplementary R package:** waveform_1.0.1.tar.gz.

Table S1 **The gene ontology enrichment for Figure 4.** For GO ontology analysis, the genes in each cohort identified in Figure 4 out of the 5570 genes contained by the dataset were checked for enrichment using the package GOstats (Falcon and Gentleman, 2007).

## References

[B1] Ahdesmäki M. Lähdesmäki H. Pearson R. Huttunen H. Yli-Harja O. (2005). Robust detection of periodic time series measured from biological systems. BMC Bioinformatics 6:117. 10.1186/1471-2105-6-117 15892890 PMC1168888

[B2] Alter O. Brown P. O. Botstein D. (2003). Generalized singular value decomposition for comparative analysis of genome-scale expression data sets of two different organisms. Proc. Natl. Acad. Sci. U.S.A. 100, 3351–3356. 10.1073/pnas.0530258100 12631705 PMC152296

[B3] Amariei C. Machné R. Sasidharan K. Gottstein W. Tomita M. Soga T.. (2013). The dynamics of cellular energetics during continuous yeast culture. Conf. Proc. Annu. Int. Conf. IEEE Eng. Med. Biol. Soc. 2013, 2708–2711. 10.1109/EMBC.2013.6610099 24110286

[B4] Aon M. A. Cortassa S. O'Rourke B. (2004). Percolation and criticality in a mitochondrial network. Proc. Natl. Acad. Sci. U.S.A. 101, 4447–4452. 10.1073/pnas.0307156101 15070738 PMC384767

[B5] Aon M. A. Roussel M. R. Cortassa S. O'Rourke B. Murray D. B. Beckmann M.. (2008). The scale-free dynamics of eukaryotic cells. PLoS ONE 3:e3624. 10.1371/journal.pone.0003624 18982073 PMC2575856

[B6] Calza S. Valentini D. Pawitan Y. (2008). Normalization of oligonucleotide arrays based on the least-variant set of genes. BMC Bioinformatics 9:140. 10.1186/1471-2105-9-140 18318917 PMC2324100

[B7] de Lichtenberg U. Jensen L. J. Fausbøll A. Jensen T. S. Bork P. Brunak S. (2005). Comparison of computational methods for the identification of cell cycle-regulated genes. Bioinformatics 21, 1164–1171. 10.1093/bioinformatics/bti093 15513999

[B8] Dowse H. B. (2007). Statistical analysis of biological rhythm data. Methods Mol. Biol. 362, 29–45. 10.1007/978-1-59745-257-1_2 17416999

[B9] Falcon S. Gentleman R. (2007). Using GOstats to test gene lists for GO term association. Bioinformatics 23, 257–258. 10.1093/bioinformatics/btl567 17098774

[B10] Gibbison B. Angelini G. D. Lightman S. L. (2013). Dynamic output and control of the hypothalamic-pituitary-adrenal axis in critical illness and major surgery. Br. J. Anaesth. 111, 347–360. 10.1093/bja/aet077 23661405

[B11] Johnson C. H. Egli M. (2014). Metabolic compensation and circadian resilience in prokaryotic cyanobacteria. Annu. Rev. Biochem. 83, 221–247. 10.1146/annurev-biochem-060713-035632 24905782 PMC4259047

[B12] Kageyama R. Niwa Y. Isomura A. González A. Harima Y. (2012). Oscillatory gene expression and somitogenesis. Wiley Interdiscip. Rev. Dev. Biol. 1, 629–641. 10.1002/wdev.46 23799565

[B13] Klevecz R. R. Bolen J. Forrest G. Murray D. B. (2004). A genomewide oscillation in transcription gates DNA replication and cell cycle. Proc. Natl. Acad. Sci. U.S.A. 101, 1200–1205. 10.1073/pnas.0306490101 14734811 PMC337030

[B14] Klevecz R. R. Li C. M. (2007). Evolution of the clock from yeast to man by period-doubling folds in the cellular oscillator. Cold Spring Harb. Symp. Quant. Biol. 72, 421–429. 10.1101/sqb.2007.72.040 18419300 PMC2671296

[B15] Klevecz R. R. Murray D. B. (2001). Genome wide oscillations in expression. Wavelet analysis of time series data from yeast expression arrays uncovers the dynamic architecture of phenotype. Mol. Biol. Rep. 28, 73–82. 10.1023/A:1017909012215 11931391

[B16] Kyriacou C. P. (2009). Clocks, cryptochromes and Monarch migrations. J. Biol. 8, 55. 10.1186/jbiol153 19591650 PMC2737371

[B17] Lehmann R. Machné R. Georg J. Benary M. Axmann I. Steuer R. (2013). How cyanobacteria pose new problems to old methods: challenges in microarray time series analysis. BMC Bioinformatics 14:133. 10.1186/1471-2105-14-133 23601192 PMC3679775

[B18] Levine J. H. Lin Y. Elowitz M. B. (2013). Functional roles of pulsing in genetic circuits. Science 342, 1193–1200. 10.1126/science.1239999 24311681 PMC4100686

[B19] Li C. M. Klevecz R. R. (2006). A rapid genome-scale response of the transcriptional oscillator to perturbation reveals a period-doubling path to phenotypic change. Proc. Natl. Acad. Sci. U.S.A. 103, 16254–16259. 10.1073/pnas.0604860103 17043222 PMC1613231

[B20] Ljung G. M. Box G. E. P. (1978). On a measure of lack of fit in time series models. Biometrika 65, 297–303. 10.1093/biomet/65.2.2978303330

[B21] Lloyd D. (2008). Intracellular time keeping: epigenetic oscillations reveal the functions of an ultradian clock, in Ultradian Rhythms from Molecules to Mind, eds. Lloyd D. Rossi E. L. (Dordrecht: Springer) 5–22

[B22] Lloyd D. Aon M. A. Cortassa S. (2001). Why homeodynamics, not homeostasis? ScientificWorldJournal 1, 133–145. 10.1100/tsw.2001.20 12805697 PMC6084724

[B23] Lloyd D. Murray D. B. (2005). Ultradian metronome: timekeeper for orchestration of cellular coherence. Trends Biochem. Sci. 30, 373–377. 10.1016/j.tibs.2005.05.005 15935677

[B24] Lloyd D. Murray D. B. (2006). The temporal architecture of eukaryotic growth. FEBS Lett. 580, 2830–2835. 10.1016/j.febslet.2006.02.066 16545376

[B25] Lloyd D. Murray D. B. (2007). Redox rhythmicity: clocks at the core of temporal coherence. Bioessays 29, 465–473. 10.1002/bies.20575 17450596

[B26] Lyon D. A. (2009). The discrete fourier transform, part 4: spectral leakage. J. Object Technol. 8, 23–34. 10.5381/jot.2009.8.7.c2

[B27] Machné R. Murray D. B. (2012). The yin and yang of yeast transcription: elements of a global feedback system between metabolism and chromatin. PLoS ONE 7:e37906. 10.1371/journal.pone.0037906 22685547 PMC3369881

[B28] Murray D. B. Beckmann M. Kitano H. (2007). Regulation of yeast oscillatory dynamics. Proc. Natl. Acad. Sci. U.S.A. 104, 2241–2246. 10.1073/pnas.0606677104 17284613 PMC1794218

[B29] Murray D. B. Klevecz R. R. Lloyd D. (2003). Generation and maintenance of synchrony in *Saccharomyces cerevisiae* continuous culture. Exp. Cell Res. 287, 10–15. 10.1016/S0014-4827(03)00068-5 12799177

[B30] Murtagh F. (1985). Multidimensional Clustering Algorithms. COMPSTAT Lectures 4. Wuerzburg: Physica-Verlag

[B31] Oppenheim A. V. Schafer R. W. Buck J. R. (1999). Discrete Time Signal Processing. New Jersey: Prentice Hall

[B32] Prasad S. Bruce L. M. (2008). Limitations of principal components analysis for hyperspectral target recognition. IEEE Geosci. Remote Sens. Lett. 5, 625–629. 10.1109/LGRS.2008.2001282

[B33] Raychaudhuri S. Stuart J. M. Altman R. B. (2000). Principal components analysis to summarize microarray experiments: application to sporulation time series. Pac. Symp. Biocomput. 455–66. 10902193 10.1142/9789814447331_0043 PMC2669932

[B34] Salgado E. Murray D. B. Lloyd D. (2002). Some antidepressant agents (Li+, monoamine oxidase type A inhibitors) perturb the ultradian clock in *Saccharomyces cerevisiae*. Biol. Rhythm Res. 33, 351–361. 10.1076/brhm.33.3.351.8256

[B35] Salomon R. M. Cowan R. L. (2013). Oscillatory serotonin function in depression. Synapse 67, 801–820. 10.1002/syn.21675 23592367 PMC3786873

[B36] Salvatore P. Indic P. Murray G. Baldessarini R. J. (2012). Biological rhythms and mood disorders. Dialogues Clin. Neurosci. 14, 369–379. 23393414 10.31887/DCNS.2012.14.4/psalvatore PMC3553575

[B37] Sasidharan K. Amariei C. Tomita M. Murray D. B. (2012a). Rapid DNA, RNA and protein extraction protocols optimized for slow continuously growing yeast cultures. Yeast 29, 311–322. 10.1002/yea.2911 22763810

[B38] Sasidharan K. Soga T. Tomita M. Murray D. B. (2012b). A yeast metabolite extraction protocol optimised for time-series analyses. PLoS ONE 7:e44283. 10.1371/journal.pone.0044283 22952947 PMC3430680

[B39] Sasidharan K. Tomita M. Aon M. Lloyd D. Murray D. B. (2012c). Time-structure of the yeast metabolism *in vivo*. Adv. Exp. Med. Biol. 736, 359–379. 10.1007/978-1-4419-7210-1_21 22161340

[B40] Singleton R. (1969). An algorithm for computing the mixed radix fast Fourier transform. IEEE Trans. Audio Electroacoust. 17, 93–103. 10.1109/TAU.1969.1162042

[B41] Slavov N. Macinskas J. Caudy A. Botstein D. (2011). Metabolic cycling without cell division cycling in respiring yeast. Proc. Natl. Acad. Sci. U.S.A. 108, 19090–19095. 10.1073/pnas.1116998108 22065748 PMC3223432

[B42] Sobie E. A. (2011). Bistability in biochemical signaling models. Sci. Signal. 4, tr10. 10.1126/scisignal.2001964 21954291 PMC4118931

[B43] Song J. Z. Duan K. M. Ware T. Surette M. (2007). The wavelet-based cluster analysis for temporal gene expression data. EURASIP J. Bioinform. Syst. Biol. 2007, 39382. 10.1155/2007/39382 17713589 PMC3171337

[B44] Strimmer K. (2008). fdrtool: a versatile R package for estimating local and tail area-based false discovery rates. Bioinformatics 24, 1461–1462. 10.1093/bioinformatics/btn209 18441000

[B45] Team R. D. C. (2008). R: A Language and Environment for Statistical Computing. Available at: http://www.r-project.org

[B46] Venables W. N. Ripley B. D. (2002). Modern Applied Statistics with S, 4th Edn. New York, NY: Springer. 10.1007/978-0-387-21706-2

[B47] Wang D. Arapostathis A. Wilke C. O. Markey M. K. (2012). Principal-oscillation-pattern analysis of gene expression. PLoS ONE 7:e28805. 10.1371/journal.pone.0028805 22253697 PMC3254616

[B48] Ward J. H. (1963). Hierarchical grouping to optimize an objective function. J. Am. Stat. Assoc. 58, 236–244. 10.1080/01621459.1963.10500845

[B49] Yamada R. Ueda H. R. (2007). Microarrays: statistical methods for circadian rhythms. Methods Mol. Biol. 362, 245–264. 10.1007/978-1-59745-257-1_17 17417014

